# Cardiovascular health and workload in university workers

**DOI:** 10.47626/1679-4435-2023-1211

**Published:** 2024-11-14

**Authors:** Horrana Carolina Bahmad Gonçalves, Pedro Henrique de Almeida Silva, Viviane Soares

**Affiliations:** 1 Medical School, Universidade Evangélica de Goiás (UniEVANGÉLICA), Anápolis, GO, Brazil; 2 Graduate Program in Human Movement and Rehabilitation, UniEVANGÉLICA, Anápolis, GO, Brazil

**Keywords:** cardiovascular system, workload, cardiometabolic risk factors, universities, sistema cardiovascular, carga de trabalho, fatores de risco cardiometabólico, universidades

## Abstract

**Introduction:**

High workloads contribute to the development of risk factors for cardiovascular disease. One contributing factor is the difficulty in minimizing the effects of work overload on activities of daily living.

**Objectives:**

To determine whether there is an association between workload and cardiovascular health among employees of a higher education institution.

**Methods:**

An analytical cross-sectional study of 121 employees. Workload was measured by a self-report questionnaire. Cardiovascular health was assessed using seven measures (diet, physical activity, body mass index, smoking, systolic and diastolic blood pressure, fasting glucose, and total cholesterol). The International Physical Activity Questionnaire and the Mediterranean Diet Questionnaire were used to assess physical activity and diet, respectively.

**Results:**

71 women (58.7%) and 50 men (41.3%) participated. Employees working > 40 hours/week (44.6%) had higher body mass index (∆ = +2.2 kg/m^2^, p = 0.015), blood pressure (systolic blood pressure: ∆ = +8.6 mmHg, p = 0.002; diastolic blood pressure: ∆ = +4.1 mmHg; p = 0.032) and lower cardiovascular health score (∆= -1.1; p = 0.009). There was a positive correlation between working hours and body mass index (p = 0.013) and systemic blood pressure (p = 0.08), and a negative correlation for cardiovascular health score (p = 0.047).

**Conclusions:**

Workers with a workload > 40 hours/week may be susceptible to worse cardiovascular health, especially in terms of obesity and systemic blood pressure.

## Abstract (pt)

**Introdução:**

A carga horária de trabalho elevada contribui para o desenvolvimento de fatores de risco para doenças cardiovasculares. Um fator que contribui para isso é a dificuldade de minimizar os efeitos da sobrecarga de trabalho sobre as atividades de vida diária.

**Objetivos:**

Verificar se há relação entre a carga horária de trabalho e a saúde cardiovascular de colaboradores de uma instituição de ensino superior.

**Métodos:**

Foi realizado um estudo transversal analítico com a participação de 121 colaboradores. A carga horária de trabalho foi mensurada por um questionário de autorrelato. A saúde cardiovascular foi avaliada por sete métricas (dieta, nível de atividade física, índice de massa corporal, tabagismo, pressão arterial sistólica e pressão arterial diastólica, glicemia de jejum e colesterol total). O Questionário Internacional de Atividade Física e o da Dieta do Mediterrânea foram utilizados para avaliar o nível de atividade física e dieta, respectivamente.

**Resultados:**

Participaram do estudo 71 mulheres (58,7%) e 50 homens (41,3%). Os colaboradores com carga horária de trabalho > 40 h/sem (44,6%) apresentaram valores superiores para índice de massa corporal (∆ = +2,2 kg/m^2^, p = 0,015) e pressão arterial (pressão arterial sistólica: ∆ = +8,6 mmHg, p = 0,002; pressão arterial diastólica: ∆ = +4,1 mmHg; p = 0,032) e menor escore de saúde cardiovascular (∆ = -1,1; p = 0,009). Houve correlação positiva entre a carga horária de trabalho com o índice de massa corporal (p = 0,013) e pressão arterial sistêmica (p = 0,08) e negativa para o escore de saúde cardiovascular (p = 0,047).

**Conclusões:**

Os colaboradores com carga horária de trabalho > 40 h/sem podem estar susceptíveis a uma pior saúde cardiovascular, principalmente nos indicadores de obesidade e de pressão arterial sistêmica.

## INTRODUCTION

Overtime (greater than 8 hours a day) means longer working hours, culminating in a standard 44-hour weekly workload (WL).^1,2^ Currently, most companies worldwide demand their employees to work overtime.^1^ According to the International Labor Organization (ILO), approximately 488 million people (8.9% of the world’s population) worked overtime in 2016, causing around 745,194 deaths and 23.3 million people with disabilities due to ischemic heart disease and stroke combined.^3^ These figures are alarming, given that a weekly WL less than 40 hours is associated with lower occupational risks.^3,4^

Cardiovascular diseases (CVDs) are a leading cause of death worldwide,^5^ and high WL are associated with the emergence of these diseases.^6^ The most frequent CVDs are coronary heart disease, stroke, and thrombosis.^6^ Cardiometabolic conditions such as obesity, hypertension, and diabetes are also common in workers.^3,6^ In addition to these events, long WL are associated with risk factors such as a sedentary lifestyle,^7^ poor dietary intake,^8^ smoking,^7^ high body mass index (BMI),^9^ systemic blood pressure (BP),^1,4^ and total cholesterol (TC).^9^ However, these parameters are the same as those primarily considered for cardiovascular health (CVH) assessment.^10^

CVH can be defined as the physiological and balanced functioning of the vascular and cardiac systems, unaffected by possible comorbidities that alter cardiovascular homeostasis.^10^ The American Heart Association (AHA) has defined CVH and using this term aims to reduce public health costs and increase life expectancy and quality of life (QoL) for the population by 2020. This approach represents a mindset shift from factors already in place, which curative care is concerned, to a perspective of gaining health rather than disease.

The seven parameters employed (physical activity level [PAL], diet, smoking, BMI, systemic BP, fasting glucose [FG] and TC) identify the state of CVH and help to devise prevention strategies and, consequently, reduce the prevalence of CVDs and early mortality.^10^ Currently, evidence affirms that these parameters are optimal for predicting the CVH status and fostering population health.^11^

However, studies within the academic setting focus more on students than on employees when it comes to health prevention and education.^12^ No studies have so far been found in the literature relating WL and CVH in university staff during the covid-19 pandemic. In addition, it is known that high WL is associated with CVDs and cardiometabolic disorders, in addition to leading to the emergence of risk factors such as stress, high BMI, high systemic BP, smoking, and a sedentary lifestyle.^3,4,7,9^ Therefore, this study aimed to determine whether WL and CVH are associated in university employees.

## METHODS

### SAMPLE

This is an analytical cross-sectional study conducted with employees of the Universidade Evangélica de Goiás (UniEVANGÉLICA). The university has 1,726 employees, including faculty, administrative, and general service staff. The survey was collected between January and June 2021 in all of the shifts (morning, afternoon, and evening). A total of 302 of the 1,726 employees were invited to participate in the study. Of these 302, only 133 agreed to participate, 12 of whom were excluded due to their continued use of medication for hypertension and diabetes mellitus. The study included employees who had been employed since 2019 and were aged between 18 and 59. Those with a clinical diagnosis of chronic diseases such as chronic obstructive pulmonary disease, CVDs, and those with cognitive impairment were excluded.

The sample was calculated using the G*Power (version 3.1, Universität Dusseldorf, Germany), considering the comparison test between groups (Student’s t-test) and multiple linear regression, with a sample power of 95%, an average effect size of 0.15, and a significance level of 5%, thus requiring 119 employees.

### STUDY DESIGN

Sociodemographic data were collected by appointment according to employees’ availability and the Short Form-36 (SF-36), Mediterranean Diet, and International Physical Activity Questionnaires short form (IPAQ-SF) surveys were completed. Blood samples were only collected in the morning by a laboratory specializing in clinical analysis. In addition, these procedures were individualized in an air-conditioned and reserved room to reduce the risk of embarrassment among employees and the risk of covid-19 contamination.

### SOCIODEMOGRAPHIC DATA

The participants filled in an identification form with information such as age, sex, educational background (elementary school, unfinished high school, high school, college degree, and graduate studies), monthly income (up to two, two to three, or more than three Brazilian minimum wages), associated comorbidities, and continued use of medication.

### WORKLOAD

WL was assessed using a self-report questionnaire including information on daily WL, starting and finishing time, and meal breaks. The questionnaire was classified following Lee et al.^13^ Thus, the sample was divided into two groups, the first with a weekly WL up to 40 hours and the second with a weekly WL greater than 40 hours.

### CARDIOVASCULAR HEALTH

The CVH was assessed with the AHA recommendations, namely, seven factors to predict the CVH status in adults, four behavioral (diet, PAL, smoking, and BMI) and three biological (systemic BP, FG, TC).^10^ These parameters were categorized as poor, moderate, and optimal, corresponding to 0, 1, and 2 points, respectively (Chart 1). When these points were added up, CVH was classified as poor (0 to 8 points), moderate (9 to 10 points), and optimal (11 to 14 points).

**Chart 1:** Parameters of CVH according to the American Heart Association

CVH	Poor (0-8 points)	Moderate (9-10 points)	Optimal (11-14 points)
Smoking	Current smoker	Former smoker	Never smoked
Diet	Points (0-22)	Points (23-34)	Points (35-55)
PAL	None (0 min/week)	< 149 min MPA/week or < 74 min VPA/week or 1-49 min MPA + VPA	≥ 150 min MPA/week or ≥ 75 min VPA/week or ≥ 150 min de MPA + VPA
BMI	≥ 30 kg/m^2^	25-29.99 kg/m^2^	< 25 kg/m^2^
Blood glucose	≥ 126 mg/dL	100-125 mg/dL	< 100 mg/dL
TC	≥ 240 mg/dL	200-239 mg/dL	< 200 mg/dL
BP	≥ 140/90 mmHg	120-139/80-89 mmHg	< 120/80 mmHg

TC = total cholesterol; BMI = body mass index; MPA/week = moderate physical activity/week; PAL = physical activity level; BP = blood pressure; CVH = cardiovascular health; VPA/week = vigorous physical activity/week.

According to the AHA, the diet and PAL should be adapted according to the characteristics of the country where the study is being conducted.^10^ The dietary factors were adapted according to the Manual de Alimentação Cardioprotetora (Handbook of Cardioprotective Nutrition) developed by the Brazil Ministry of Health and the Hospital do Coração (HCor) for the Brazilian population.^14^ The food groups presented in this manual are the same ones found in the Mediterranean Diet score that was used in this study.^15^ This survey consists of 11 items (unrefined cereals, fruit, vegetables, potatoes, legumes, olive oil, fish, beef, poultry, whole milk products, and alcohol) which are graded for adherence. In addition, the score ranges from 0 to 55 points and the higher it is, the more cardioprotective and healthier the diet is.^15^ Thus, the scores were adjusted from the degree of adherence to classify CVH (Chart 1).

PAL was measured using the IPAQ-SF questionnaire with information on the frequency and duration of physical activity in different intensities (moderate, vigorous) and daily activities/leisure, calculated in minutes per week (min/week).^16^ The optimal CVH for PAL was defined when employees performed 150 min/week of moderate physical activity (MPA) or 75 min/week of vigorous physical activity (VPA). Moderate was considered when the worker performed physical activity for 1-49 min/week and poor when they did not exercise at all during the week.

A self-report questionnaire was used to assess smoking (current smoker, former smoker, never smoked). BMI was defined by calculating (weight [kg]/height [m^2^]), a digital scale (G-Tech, model Balgl10, São Paulo, Brazil) and a portable stadiometer (Sanny, São Paulo, Brazil) were used to measure body mass and height, respectively. The reference values^17^ for BMI were: optimal when less than 25 kg/m^2^, moderate between 25 and 29.9 kg/m^2^, and poor when greater than or equal to 30 kg/m^2^.

For serum glucose and TC, fasting for 8-12 hours was recommended using the enzymatic colorimetric method. Diastolic BP (DBP) and systolic BP (SBP) were measured using a semi-automatic device (OMRON, model HEM 705CP, Kyoto, Japan). Optimal TC was considered to be less than 200 mg/dL, moderate between 200-239 mg/dL, and poor greater than or equal to 240 mg/dL. For blood glucose, less than 100 mg/dL was considered optimal, between 100-125 mg/dL moderate, and greater than or equal to 126 mg/dL poor.^18^

### DATA ANALYSIS

The results were expressed as means, standard deviations, frequencies, percentages, and charts. The normality of the data was checked using Student’s Kolmogorov-Smirnov (KS) test. Student’s t-test for independent samples (symmetrical distribution) and the Mann-Whitney test (asymmetrical distribution) were used to compare the groups (less than or equal to 40 hours per week and greater than 40 hours per week). The chi-square test was used to associate WL with the CVH score and CVH parameters. Spearman’s correlation coefficient was used to correlate WL with CVH parameters. The p-value was less than 0.05, and the data were analyzed using the Statistical Package for Social Science (SPSS, IBM, version 23, Armont, NY).

### ETHICAL ASPECTS

All employees who participated in this study signed an informed consent form. The study complied with Resolution No. 466/12 of the Conselho Nacional de Saúde (CNS, Brazil National Health Council) and the Ethics and Research Committee of UniEVANGÉLICA approved the study No. 4.512.382/2021.

## RESULTS

The sociodemographic characteristics of the sample are described in Table 1. The study involved 121 university employees. As for the WL, 55.4% of the employees worked less than or equal to 40 hours per week and 45% worked more than 40 hours per week. Employees with WL less than or equal to 40 hours per week had lower BMI values (∆ = - 8.9 kg, p = 0.006). When sex was assessed, 57.4% were female and worked more than 40 hours per week.

Employees with WL less than 40 hours per week had higher BMI (∆ = +2.2 kg/m^2^, p = 0.015), SBP (∆ = +8.6 mmHg, p = 0.002) and DBP (∆ = +4.1 mmHg; p = 0.032), while the number of parameters at optimal levels (∆ = -0.6; p = 0.009) and CVH score (∆ = -1.1; p = 0.009) were lower (Table 2).

**Table 1:** Sociodemographic characteristics of university employees (n = 121)

Sociodemographic data	Total (n = 121)	WL		p-value^*^
		≤ 40 h/week (n = 67)	> 40 h/week (n = 54)	
	Mean (SD)	Mean (SD)	Mean (SD)	
Age (years)	35.97 (10.13)	34.81 (10.16)	37.41 (10.01)	0.161
Body mass (kg)	72.77 (17.86)	68.79 (16.27)	77.70 (18.65)	0.006
Height (cm)	1.67 (0.10)	1.7 (0.09)	1.7 (0.11)	0.139
Sociodemographic data	n (%)	n (%)	n (%)	p-value†
Sex				
Female	71 (58.7)	48 (71.6)	23 (42.6)	0.001
Male	50 (41.3)	19 (28.4)	31 (57.4)	
Education				
Elementary school	4 (3.3)	3 (4.5)	1 (1.9)	
High school	20 (16.5)	8 (11.9)	12 (22.2)	
Unfinished high school	29 (24.0)	19 (28.4)	10 (18.5)	0.432
College degree	32 (26.4)	17 (25.4)	15 (27.8)	
Graduate studies	36 (29.8)	20 (29.9)	16 (29.6)	
Comorbidities				
Yes	17 (14.0)	11 (16.4)	6 (11.1)	0.404
No	104 (86.0)	56 (83.6)	48 (88.9)	
Medication				
Antidepressants/anxiolytics	19 (15.7)	12 (17.9)	7 (13)	
Other	15 (12.4)	13 (19.4)	2 (3.7)	0.017
No medication	90 (71.9)	42 (62.7)	45 (83.3)	
Monthly income (minimum wage)		-	-	
Up to two	50 (41.3)	30 (44.8)	20 (37)	
two to three	41 (33.9)	25 (37.3)	16 (29.6)	0.148
More than three	30 (24.8)	12 (17.9)	18 (33.3)	

WL = workload; SD = standard deviation.

* Student’s t-test or Mann-Whitney test.

† Chi-squared test for independence. Data for p < 0.05.

**Table 2:** Comparison of weekly WL with the number of factors, parameters, and CVH scores (n = 121)

CVH	Total (n = 121)	CVH		p-value^*^
		≤ 40 h/week (n = 67)	> 40 h/week (n = 54)	
Behavioral				
Smoking	n (%)	n (%)	n (%)	
Current smoker	06 (5.0)	03 (4.5)	03 (5.6)	
Former smoker	02 (1.7)	0 (0)	02 (3.7)	0.185
Never smoked	113 (93.3)	64 (95.5)	49 (90.7)	
	Mean (SD)	Mean (SD)	Mean (SD)	p-value†
PAL				
Min/week	245.87 (203.47)	242.5 (186.9)	250.0 (224.0)	0.804
Min/day	35.12 (29.07)	34.6 (26.7)	35.7 (32.0)	0.804
Diet				
Points	27.87 (5.71)	28.5 (5.5)	27.0 (5.9)	0.152
BMI (kg/m^2^)	25.97 (5.07)	25.0 (4.9)	27.2 (5.0)	0.015
Biological				
SBP (mmHg)	123.37 (15.28)	119.5 (14.4)	128.1 (15.1)	0.002
DBP (mmHg)	80.77 (11.01)	79.0 (10.1)	83.1 (11.7)	0.032
FG (mg/dL)	86.36 (20.23)	83.8 (8.2)	89.5 (28.7)	0.337
TC (mg/dL)	181.96 (40.36)	176.3 (39.7)	189.0 (40.5)	0.804
CVH score	9.89 (2.14)	10.4 (1.7)	9.3 (2.4)	0.009
Number of optimal parameters	4.95 (1.07)	5.2 (0.9)	4.6 (1.2)	0.009

WL = workload; TC = total cholesterol; SD = standard deviation; FG = fasting glucose; BMI = body mass index; PAL = physical activity level; DBP = diastolic blood pressure; SBP = systolic blood pressure; CVH = cardiovascular health.

* Chi-square test for independence.

† Student’s t-test or Mann-Whitney test. Data for p < 0.05.

**Figure 1:**
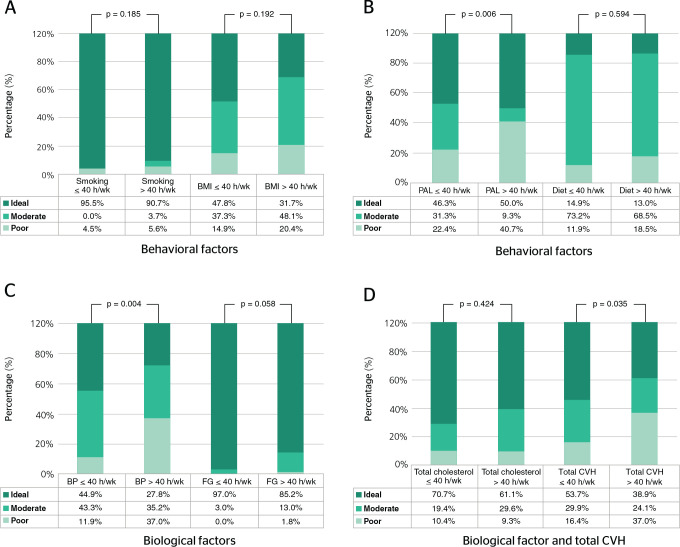
Association between workload (WL) and cardiovascular health (CVH) factors and total CVH (chi-square test). Data calculated for p less than 0.05. A) WL × smoking; WL × body mass index (BMI); B) WL × physical activity level (PAL); WL × diet; C) WL × systemic blood pressure (BP); WL × fasting glucose (FG); D) WL × total cholesterol (TC); WL × total CVH.

The categorical association of WL with the seven CVH parameters indicated a significant association with PAL (p = 0.006), systemic BP (p = 0.004), and total CVH (p = 0.035) (Figure 1).

Weekly WL was positively correlated with BMI (p = 0.013) and systemic BP (p = 0.08), while it was negatively correlated with the CVH score (p = 0.047) (Table 3). It is suggested that the lower the WL, the more optimal the CVH of university employees.

**Table 3:** Correlation of weekly WL with CVH score and parameters (n = 121)

Variables	Weekly WL	
	r	p-value^*^
BMI (kg/m^2^)	0.225	0.013
SBP (mmHg)	0.239	0.008
CVH score	- 0.181	0.047

WL = workload; BMI = body mass index; SBP = systolic blood pressure;

CVH = cardiovascular health.

* Spearman’s correlation coefficient. Value considered for p < 0.05.

## DISCUSSION

This study has shown that employees with a weekly WL greater than 40 hours had higher BMI, SBP and DBP values, while the number of parameters at optimal levels and the CVH score were lower. In addition, the majority of employees with a weekly WL greater than 40 hours had moderate and poor levels of CVH parameters such as PAL and systemic BP, as well as total CVH. Also, a positive correlation was found between weekly WL with BMI and SBP, and a negative correlation for the CVH score.

The results showed that employees with a weekly WL greater than 40 hours had higher values for BMI and systemic BP (SBP and DBP). These findings were similar to those of a meta-analysis which, while not assessing CVH, suggested that long working hours are associated with obesity, smoking, a sedentary lifestyle, hypertension, and impaired cardiac function.^6^ As a result, cardiovascular events such as coronary heart disease, strokes, and deep vein thrombosis are associated.^6,13^

The employees evaluated had their parameters at optimal levels and a lower CVH score when they were classified as having a WL greater than 40 hours per week. It is known that CVH at optimal levels is essential for preventing chronic noncommunicable diseases and mitigating the risk factors they are associated with.^19^ Additionally, it helps to increase life expectancy and reduce all-cause mortality.^19^ As seen in this study, the behavioral and biological factors (BMI and systemic BP) of CVH^9^ were high, suggesting that a weekly WL greater than 40 hours,^1,4,9,20^ can be harmful to the cardiovascular system when not balanced with healthy habits.

It is worth noting that, in addition to long working hours, the environment (physical and interpersonal relationships) and perception of one’s own life contribute to reducing CVH. In addition to these factors, others are also associated with reduced CVH, such as low QoL^21^ and increased liver enzymes, which may be related to stress.^22,23^

As for the categorical association, the majority of employees who had a WL greater than 40 hours per week had CVH parameters (PAL and systemic BP) and total CVH at moderate and poor levels. Evidence suggests that people who work longer hours a week are more likely to have systemic arterial hypertension, occupational stress, and low PAL.^7,24,25^

Studies report that long working hours have a direct relationship with occupational stress^21^ and C-reactive protein,^20^ and increase twofold the risk of coronary heart disease,^21^ with data adjusted for age and sex.^20,21^ This study found a positive correlation between weekly WL and BMI and systemic BP parameters, and a negative correlation for the CVH score; however, the data was not analyzed according to sex and age. In addition to the WL, men work more overtime, which means they are more likely to develop CVDs.^22^

This study has some limitations. Although the sample had a power of inference, employees at the university were less likely to participate in this study due to the covid-19 pandemic. In addition, no cause-effect relationship was established due to the type of study. Lastly, occupational stress was not collected, although this is a relevant variable that could be correlated with CVH or WL. As for the positive aspects of the study, the seven CVH parameters were evaluated in accordance with the AHA, and adaptation to diet and PAL following the characteristics of the Brazilian population is recommended. So far, no studies have been found relating WL to CVH in university employees during the covid-19 pandemic. This is the first study to report these findings, and more evidence is needed due to its importance in promoting health among employees.

## CONCLUSIONS

The results identified that a weekly WL greater than 40 hours can increase BMI and systemic BP, while reducing the number of parameters at optimal levels and the CVH score. WL had a positive correlation with BMI and systemic BP parameters of the CVH, while it was negative with the CVH score. It is suggested that preventive health programs be implemented within universities to encourage employees to engage in healthy behaviors, such as proper nutrition and physical activity. These strategies are essential for achieving the ideal CVH parameters.
